# Interactive effects of molecular subtypes with tumor size and extracranial metastatic pattern on risk of brain metastasis in breast cancer patients: A population‐based study

**DOI:** 10.1002/cam4.5425

**Published:** 2022-11-09

**Authors:** Bo Shen, Jieqing Li, Mei Yang, Kangkang Liu, Junsheng Zhang, Weiping Li, Yi Zhang, Kun Wang

**Affiliations:** ^1^ Shantou University Medical College Shantou China; ^2^ Department of Breast Cancer, Cancer Center Guangdong Provincial People's Hospital, Guangdong Academy of Medical Sciences Guangzhou China; ^3^ Department of Research Center for Medicine The Eighth Affiliated Hospital, Sun Yat‐Sen University Shenzhen China; ^4^ State Key Laboratory of Oncology in South China, Collaborative In‐novation Center for Cancer Medicine Sun Yat‐sen University Cancer Center Guangzhou China; ^5^ The First Affiliated Hospital/School of Clinical Medicine of Guangdong Pharmaceutical University Guangzhou China

**Keywords:** brain metastasis, breast cancer, extracranial metastasis, interaction analysis, molecular subtype, tumor size

## Abstract

**Background:**

Early detection of brain metastasis (BM) is essential for prognostic improvement in breast cancer (BC) patients. The aim was to identify predictors of BCBM in different molecular subtypes on a population‐based level.

**Methods:**

The Surveillance, Epidemiology, and End Results database was used to select BC patients diagnosed from 2010 to 2018. We evaluated the incidence and risk factors of BCBM and tested the interaction effects between molecular subtypes and other risk factors.

**Results:**

Among the 527,525 selected patients, molecular subtypes significantly interacted with T stage and extracranial metastasis (ECM) patterns on the risk of BM in the whole BC population (interaction *p* = 0.002, <0.001, respectively) and after excluding patients with unknown states of key factors. BM development was independent of the T stage only in HR−/HER2− patients (trend *p* = 0.126). We selected BC patients with single‐organ ECM and found a significant interaction between molecular subtypes and ECM patterns (interaction *p* = 0.013). The impact of ECM patterns on the risk of BM was limited to HR−/HER2− patients (trend *p* < 0.001), for whom using bone metastasis as a reference, lung metastasis increased the risk of BM (OR = 1.936, 95% CI: 1.300–2.882, *p* = 0.001).

**Conclusion:**

T stage and ECM patterns had different associations with BM in different molecular subtypes. HR−/HER2− BC had distinct features on BM development, manifested as a lack of tumor size effect and is associated with lung metastasis. Close surveillance for BM should be considered for HR−/HER2− BC patients.

## Graphical abstract

Among the 527,525 selected breast cancer patients from SEER database, we found HR−/HER2− breast cancer had distinct features on the development of brain metastasis, manifested as a lack of tumor size effect and is associated with lung metastasis. Therefore, close surveillance for brain metastasis should be considered for HR−/HER2− breast cancer patients.

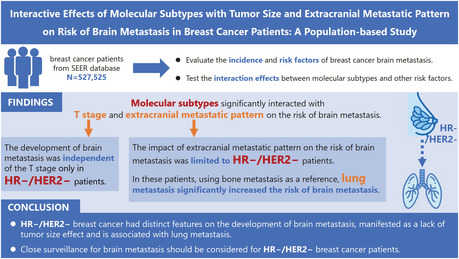

## 1 BACKGROUND

Breast cancer (BC) is currently the leading diagnosed cancer worldwide.^1^ The common sites for BC metastasis include the bone, lung, liver, and brain.^2^ An increasing incidence of brain metastasis (BM) was found in BC patients,^3^ but their prognosis remained frustrating, with the overall survival being a little over 2 years.^4^, ^5^, ^6^ Although the US National Comprehensive Cancer Network (NCCN) and European Society of Medical Oncology (ESMO) guidelines suggest brain screening be carried out only in BC patients with a known history or symptoms of BM,^7^, ^8^ one recent study found early BM detection before developing neurological symptoms could lengthen the survival of BCBM patients.^9^ Since several studies have shown improvement in the survival of BCBM patients with advances in novel systemic treatments,^10^, ^11^, ^12^ early diagnosis of BM is critical as it can provide opportunities for active treatment.

Patients at high risk of developing BM could potentially benefit from early brain screening strategies. Therefore, finding risk factors and characterizations of BCBM is important. The incidence of BM in patients with human epidermal growth factor receptor 2 (HER2)‐positive subtypes and triple‐negative breast cancer (TNBC) is remarkably higher than that of other molecular subtypes. Although data from different institutions have yielded varying results, generally, previous studies reported risk factors of BCBM included higher grade histology, large tumor size, nodal involvement, and an increasing number of extracranial metastasis (ECM) sites.^4^, ^5^, ^13^, ^14^, ^15^, ^16^, ^17^, ^18^, ^19^, ^20^, ^21^

Large tumor size and positive nodal metastasis were related to increased tumor burden, thus were traditionally believed to be correlated to the tumor's malignant capacity,^22^ which is the basis of the TNM classification system. Some researchers found that the size‐node‐metastasis relationships were not concordant with different breast subtypes. In the univariate analysis of Heitz et al.'s study, the associations of BM with tumor size or nodal status differed by immunohistochemically (IHC) defined BC subtypes.^14^ Besides, Min et al. found that tumor size had a weaker relationship with lymph node metastasis in TNBC compared with other molecular subtypes.^23^ Accordingly, it is reasonable to speculate that tumor size or nodal status may have different predive values for BM in different BC molecular subtypes.

The value of different ECM sites in predicting BM was also examined across studies, but with varying results.^4^, ^17^, ^24^, ^25^, ^26^, ^27^ Based on Paget's “seed and soil” hypothesis for nonrandom metastatic spreading,^28^ in BCBM patients, metastasis to different extracranial organs likely requires distinct traits. Since BC molecular subtypes can influence metastatic patterns as reported by many previous studies,^2^, ^29^, ^30^, ^31^ we hypothesized it may in turn influence the association between ECM patterns and BM.

To our knowledge, the interactions of molecular subtypes with tumor size, nodal status, and ECM patterns in predicting BM have not been well identified with proper statistical methods. The survival of BCBM differed by molecular subtypes.^4^, ^5^ Therefore, examining in high resolution the associations of BM with other clinicopathological factors among different molecular subtypes may not only help clarify the predictors of BCBM, gain insight into the underlying molecular basis of BC dissemination to the brain, but also guide individualized surveillance strategies for BCBM to improved survival. Therefore, the objectives of our study were to determine the predictive factors associated with BCBM and to investigate whether BC molecular subtypes can influence the associations of BM with various clinicopathological factors, especially tumor size, nodal status, and ECM patterns, by evaluating their interaction effects on a population‐based level.

## 2 METHODS

A population‐based retrospective study was conducted with data from the Surveillance, Epidemiology, and End Results (SEER) database, 9 Registries, Nov 2020 Sub (1975–2018). Selection criteria include the following: breast cancer patients diagnosed from January 1, 2010 to December 31, 2018, who were 20 years old or older, and for whom information was available regarding age, sex, race, molecular subtypes, and whether metastasis occurred to distant tissues (brain, bone, liver, or lung). Patients with carcinoma in situ and Paget's disease were excluded. Specifically, the molecular subtypes included HR+/HER2− (luminal A), HR+/HER2+ (luminal B), HR−/HER2+ (HER2), and HR−/HER2− (TNBC).

This research was based on publicly available data from the SEER database (https://seer.cancer.gov/), and a data use agreement was assigned. Analysis of de‐identified data from the SEER program was exempt from medical ethics review and no informed consent was required. All procedures performed in studies involving human participants were in accordance with the ethical standards of the 1964 Helsinki declaration and its later amendments or comparable ethical standards.

The incidence for patients with BM at diagnosis in the whole population was calculated and compared among four molecular subtypes according to age, sex, race, laterality, histological subtypes, grade, T stage, *N* stage, and ECM patterns using Pearson's Chi‐square test. Univariate and multivariate logistic regression analyses were performed to estimate odds ratios (ORs) and 95% confidence intervals (CIs) for analyses of potential risk factors of BM. Interactive effects between molecular subtypes and any other suspected risk factors were evaluated using a multiplicative interaction term and after adjusting for all covariates. Two sensitivity analyses were performed: one in which we excluded patients with unknown T stage, *N* stage, grade, or laterality, or with an imprecise classification of histological subtypes in the whole BC population to investigate the robustness of the interactive effects and another in which we exclusively included patients with only one ECM site to evaluate the stability of the relationship between BM and ECM patterns. After the statistical interaction was found, we performed stratified analysis to further test the relationship between the suspected risk factor and BM in each molecular subtype, and *p* for trend was calculated in the following multivariate logistic regression analyses after adjusting for all covariates. It was considered statistically significant when *p* values were <0.05 (two‐tailed). We used SEER*Stat version 8.3.5 to generate a case listing. Statistical analyses were performed using IBM SPSS Statistics 24.0 (SPSS Inc.).

## 3 RESULTS

### 3.1 Incidence of brain metastasis according to breast subtypes

Of the 527,525 BC patients selected, 0.35% were diagnosed with BM (*n* = 1854). The incidence of BC patients with BM at diagnosis was compared among four molecular subtypes and listed in Table 1. The HR−/HER2+ and HR−/HER2− subtypes showed high rates of brain metastasis. The incidence of BCBM increased with higher grade, higher T stage, higher *N* stage, and increased number of ECM sites whatever the molecular subtype, but the magnitude of increase differed in different molecular subtypes.

**TABLE 1:** Incidence of brain metastasis according to breast subtypes in all breast cancer patients

Characteristics	HR+/HER2−			HR+/HER2+			HR−/HER2+			HR−/HER2‐			*p* value
	Brain	All	(%)	Brain	All	(%)	Brain	All	(%)	Brain	All	(%)	
All	856	389,762	0.2	348	56,098	0.6	252	23,377	1.1	398	58,288	0.7	<0.001
Age													
20–39	40	13,407	0.3	17	4657	0.4	29	1840	1.6	24	4842	0.5	<0.001
40–59	309	139,825	0.2	181	25,709	0.7	113	11,015	1.0	176	24,615	0.7	<0.001
60–79	431	193,450	0.2	140	21,607	0.6	99	8873	1.1	171	23,770	0.7	<0.001
≥80	76	43,080	0.2	10	4125	0.2	11	1649	0.7	27	5061	0.5	<0.001
Sex													
Female	843	386,340	0.2	342	55,615	0.6	251	23,342	1.1	393	58,195	0.7	<0.001
Male	13	3422	0.4	6	483	1.2	1	35	2.9	5	93	5.4	<0.001
Race													
White	661	316,573	0.2	252	43,060	0.6	187	16,891	1.1	282	41,613	0.7	<0.001
Black	139	37,001	0.4	57	6788	0.8	41	3373	1.2	90	12,152	0.7	<0.001
American Indian/Alaska Native	4	2294	0.2	3	392	0.8	2	176	1.1	5	342	1.5	<0.001
Asian or Pacific Islander	52	33,894	0.2	36	5858	0.6	22	2937	0.7	21	4181	0.5	<0.001
Laterality													
Left	413	196,344	0.2	168	28,559	0.6	121	11,984	1.0	186	29,810	0.6	<0.001
Right	408	192,661	0.2	171	27,412	0.6	114	11,312	1.0	191	28,300	0.7	<0.001
Bilateral	1	89	1.1	0	21	NA	3	14	21.4	5	23	21.7	<0.001
Unknown	34	668	5.1	9	106	8.5	14	67	20.9	16	155	10.3	<0.001
Histology													
Ductal	607	290,923	0.2	274	48,622	0.6	179	21,039	0.9	290	50,695	0.6	<0.001
Lobular	79	49,446	0.2	8	2515	0.3	7	266	2.6	6	899	0.7	<0.001
Mixed ductal and lobular	30	25,389	0.1	12	2203	0.5	7	332	2.1	6	765	0.8	<0.001
Mucinous	13	9629	0.1	0	491	NA	0	72	NA	1	58	1.7	0.009
Tubular	0	2639	NA	0	40	NA	0	1	NA	0	4	NA	NA
Metaplastic	1	589	0.2	0	48	NA	0	97	NA	12	1701	0.7	0.363
Micropapillary	3	1654	0.2	2	412	0.5	0	99	NA	0	87	NA	0.609
Adenocarcinoma	53	1356	3.9	19	334	5.7	29	231	12.6	32	424	7.5	<0.001
Papillary	1	1037	0.1	1	48	2.1	0	10	NA	0	63	NA	0.014
Medullary	0	315	NA	0	40	NA	1	61	1.6	0	538	NA	0.002
Cribriform	2	858	0.2	1	41	2.4	0	11	NA	0	16	NA	0.112
Inflammatory	5	438	1.1	6	218	2.8	10	222	4.5	11	309	3.6	0.053
Others	62	5489	1.1	25	1086	2.3	19	936	2.0	40	2729	1.5	0.008
Grade													
1	58	113,163	0.1	8	3513	0.2	1	323	0.3	4	1231	0.3	<0.001
2	331	187,334	0.2	98	22,776	0.4	49	5370	0.9	55	10,299	0.5	<0.001
≥3	264	69,809	0.4	165	26,187	0.6	129	15,575	0.8	262	43,374	0.6	<0.001
Unknown	203	19,456	1.0	77	3622	2.1	73	2109	3.5	77	3384	2.3	<0.001
T stage													
≤1	123	246,018	0.0	46	26,778	0.2	29	9929	0.3	62	24,856	0.2	<0.001
2	232	104,359	0.2	80	20,125	0.4	52	8104	0.6	92	23,056	0.4	<0.001
3	108	20,067	0.5	42	4160	1.0	26	2221	1.2	61	4809	1.3	<0.001
4	247	11,664	2.1	126	3419	3.7	90	2282	3.9	133	3925	3.4	<0.001
Unknown	146	7654	1.9	54	1616	3.3	55	850	6.5	50	1642	3.0	<0.001
*N* stage													
0	233	276,894	0.1	65	33,471	0.2	51	12,830	0.4	73	37,476	0.2	<0.001
1–2	447	98,691	0.5	197	19,570	1.0	128	8734	1.5	206	17,130	1.2	<0.001
3	98	9851	1.0	41	2283	1.8	44	1440	3.1	77	2764	2.8	<0.001
Unknown	78	4326	1.8	45	774	5.8	29	373	7.8	42	918	4.6	<0.001
Patterns of extracranial metastasis													
Bone(+) liver(−) lung(−)	267	7901	3.4	88	1473	6.0	36	428	8.4	47	820	5.7	<0.001
Bone(−) liver(+) lung(−)	24	724	3.3	15	454	3.3	16	387	4.1	9	306	2.9	0.837
Bone(−) liver(−) lung(+)	60	1361	4.4	18	392	4.6	27	344	7.8	77	762	10.1	<0.001
Bone(+) liver(+) lung(−)	75	1152	6.5	36	550	6.5	37	321	11.5	28	281	10.0	0.007
Bone(+) liver(−) lung(+)	161	2024	8.0	54	438	12.3	25	140	17.9	49	312	15.7	<0.001
Bone(−) liver(+) lung(+)	20	242	8.3	15	135	11.1	17	114	14.9	16	180	8.9	0.237
Bone(+) liver(+) lung(+)	120	812	14.8	71	356	19.9	51	197	25.9	60	216	27.8	<0.001
Bone(−) liver(−) lung(−)	129	375,546	0.0	51	52,300	0.1	43	21,446	0.2	112	55,411	0.2	<0.001
Number of extracranial metastatic sites													
0	129	375,546	0.0	51	52,300	0.1	43	21,446	0.2	112	55,411	0.2	<0.001
1	351	9986	3.5	121	2319	5.2	79	1159	6.8	133	1888	7.0	<0.001
2	256	3418	7.5	105	1123	9.3	79	575	13.7	93	773	12.0	<0.001
3	120	812	14.8	71	356	19.9	51	197	25.9	60	216	27.8	<0.001

### 3.2 Univariate and multivariate logistic regression analyses

**FIGURE 1:**
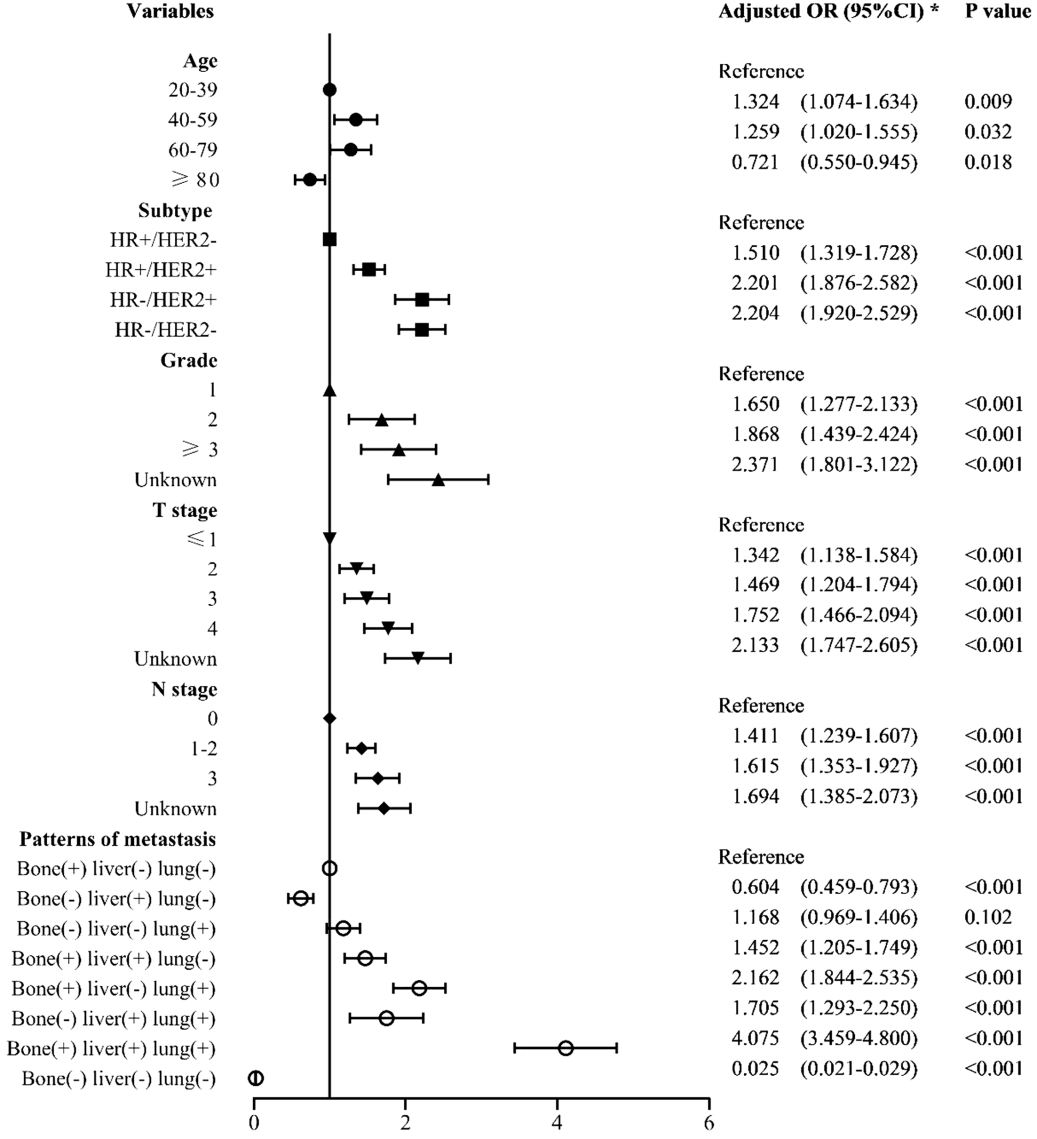
Forest plot showing the results of multivariate logistic regression analysis in all breast cancer patients. *Adjusted for age, sex, race, laterality, subtypes, histology, grade, T stage, *N* stage, and patterns of extracranial metastasis

In the univariate logistic regression analysis, the *p* values of all variables were <0.05, so all variables were incorporated into a multivariate logistic regression analysis. Multivariate logistic regression analysis among the whole population showed that age, molecular subtype, grade, T stage, *N* stage, histology, and ECM pattern were independent predictors of BM, whereas sex, race, and laterality did not significantly influence the risks of BM. HR−/HER2− was associated with the highest risk of BM (OR = 2.204, 95% CI: 1.920–2.529, *p* < 0.001) (Figure 1; Table S1).

### 3.3 Identification of interaction effects between molecular subtypes and other clinicopathological factors

After adjustment for all covariates, we found significant interactions between molecular subtypes and T stage (interaction *p* = 0.002), between molecular subtypes and *N* stage (interaction *p* = 0.002), and between molecular subtypes and patterns of ECM (interaction *p* < 0.001) that increased the risk of BCBM in the whole BC population (Table S1). After excluding patients with unknown T stage, *N* stage, grade, or laterality, or with an imprecise classification of histological subtypes, the interactions remained significant between molecular subtypes and T stage (interaction *p* = 0.035) and between molecular subtypes and patterns of ECM (interaction *p* < 0.001). However, no significant interactive effect was observed between molecular subtypes and the *N* stage (interaction *p* = 0.180), which means the association of the *N* stage with BCBM did not differ by molecular subtypes (Table S1). For patients with only one ECM site, the interaction between molecular subtypes and patterns of ECM was significant (interaction *p* = 0.013; Supplemental Table 4).

### 3.4 The association of the T stage with BM in different molecular subtypes

**FIGURE 2:**
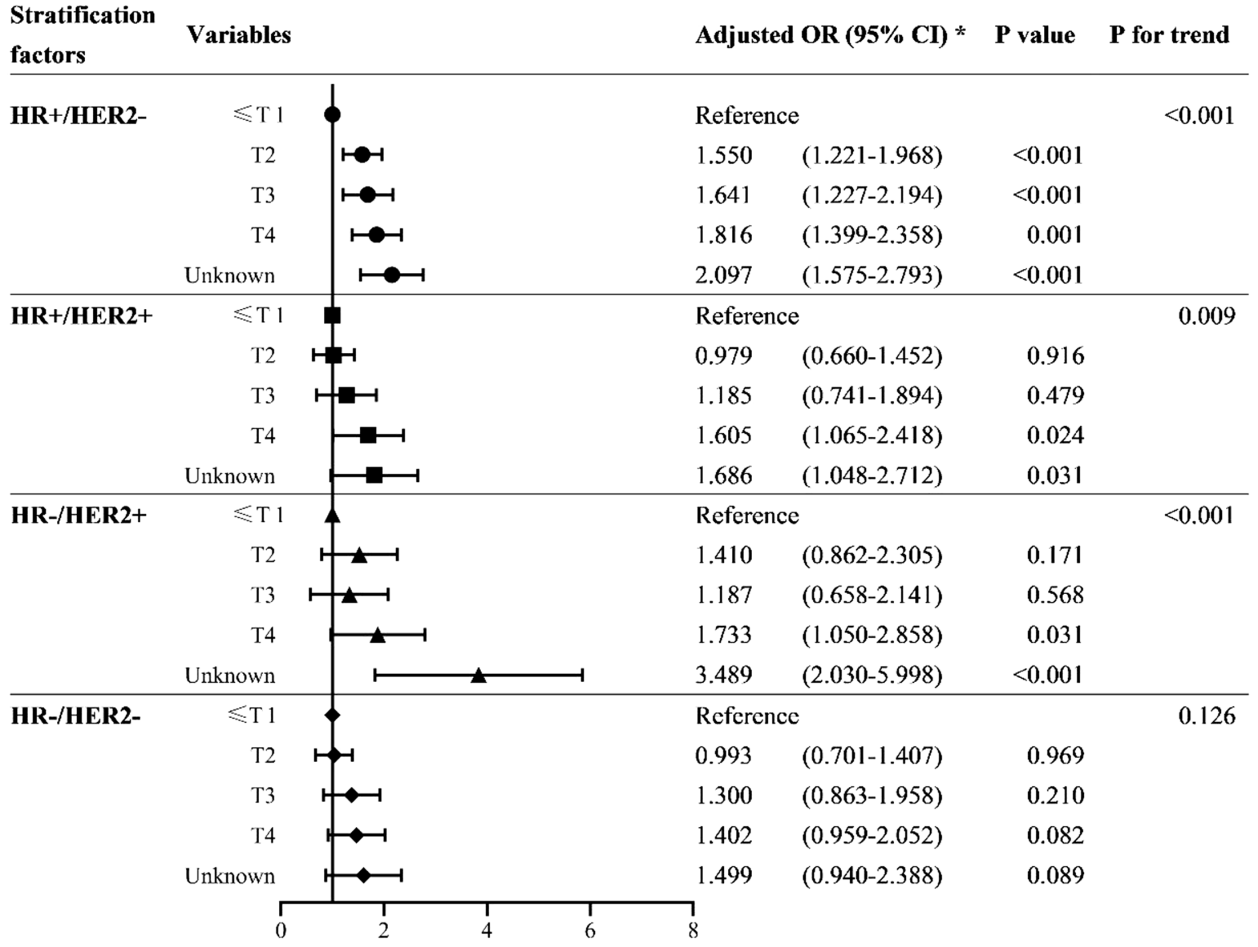
Forest plot showing stratified associations between T stage and brain metastasis by subtypes. *Adjusted for age, sex, race, laterality, histology, grade, T stage, *N* stage, and patterns of extracranial metastasis

We investigated the association of the T stage with BM stratified by molecular subtypes in the whole BC population (Figure 2; Table S1). The associations between T stage and BM were more evident in the groups with HR+/HER2− (trend *p* < 0.001), HR+/HER2+ (trend *p* = 0.009), and HR−/HER2+ (trend *p* < 0.001) than those with HR−/HER2− (trend *p* = 0.126). In HR+/HER2− patients, the larger the tumor size, the higher the risk of developing BM. In both HR+/HER2+ and HR−/HER2+ BC patients, compared with T1, only T4 significantly increased the probability of developing BM (*p* = 0.031, *p* < 0.001, respectively), whereas T2 and T3 did not significantly increase the risk of BM (*p* > 0.05 for all variables).

### 3.5 The association of the *N* stage with BM in different molecular subtypes

**FIGURE 3:**
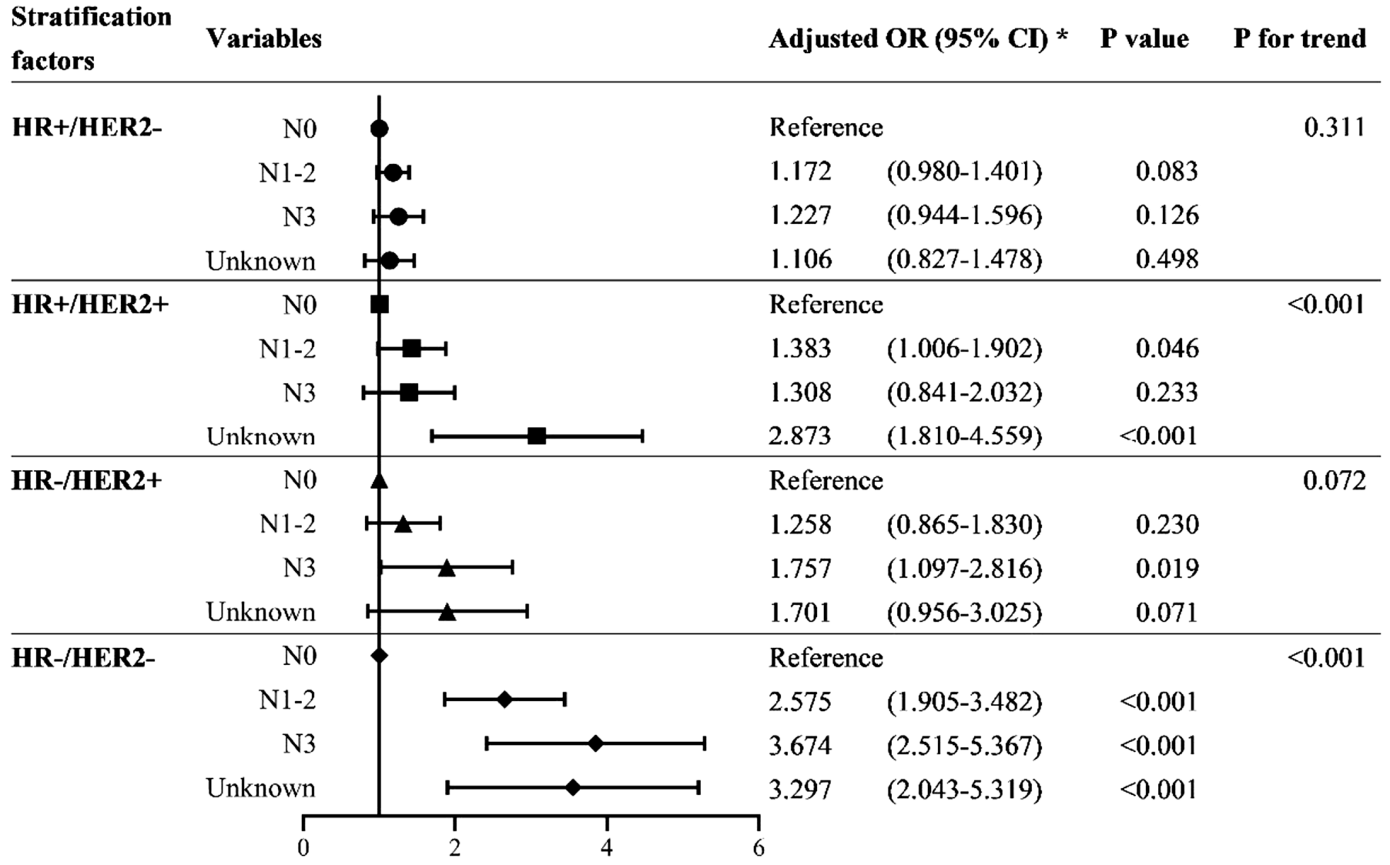
Forest plot showing stratified associations between *N* stage and brain metastasis by subtypes. *Adjusted for age, sex, race, laterality, histology, grade, T stage, *N* stage, and patterns of extracranial metastasis

We investigated the association of the *N* stage with BM stratified by molecular subtypes in the BC population (Figure 3; Table S1). Only in HR+/HER2+ and HR−/HER2− BC patients, the associations of *N* stage with BM were significant (trend *p* < 0.001 for both groups). In HR−/HER2− patients, as the extent of lymph node involvement increased from N0 to N3, the risk of BCBM increased. In HR+/HER2+ BC patients, N1‐2 is significantly associated with an increased risk of BM compared with N0 (OR, 1.383; 95% CI, 1.006–1.902, *p* = 0.046), while the risk elevation with N3 was not significant (*p* > 0.05).

### 3.6 The association of ECM patterns with BM in different molecular subtypes

**FIGURE 4:**
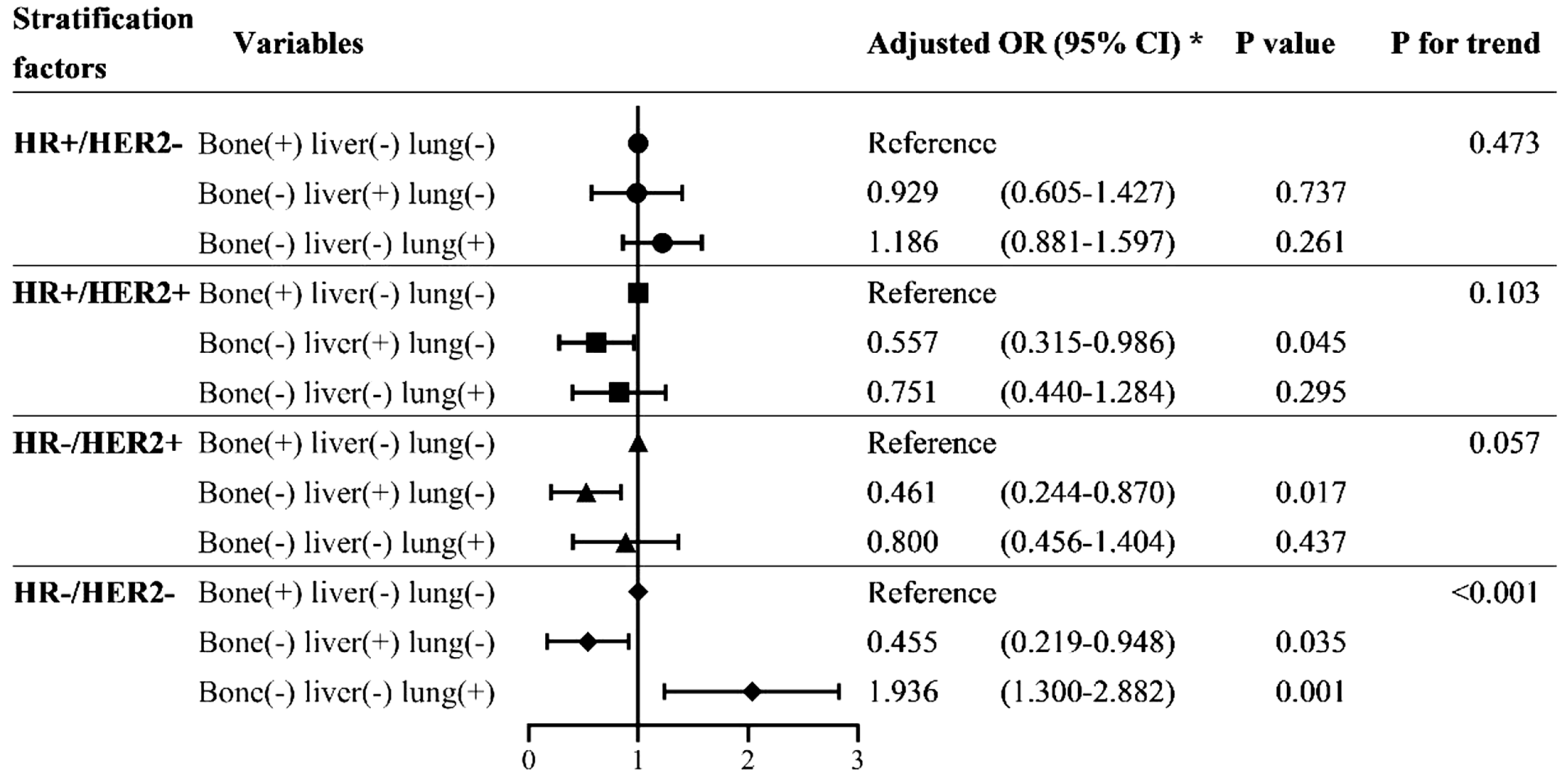
Forest plot showing stratified associations between patterns of extracranial metastasis and brain metastasis by subtypes in patients with a single extracranial metastatic site. *Adjusted for age, sex, race, laterality, histology, grade, T stage, *N* stage, and pattern of extracranial metastasis

We investigated the associations of ECM patterns with BM stratified by molecular subtypes in the BC population, which was significant across all molecular subtypes (trend *p* < 0.001 for all molecular subtypes; Table S1). However, after selecting patients with only one ECM site (*n* = 15,352), we found the association of ECM patterns with BM was significant only among HR−/HER2− BC patients (*p* for trend<0.001). For HR−/HER2− patients, using bone metastasis as a reference, lung metastasis significantly increased the risk of BM (OR = 1.936, 95% CI: 1.300–2.882, *p* = 0.001) (Figure 4; Table S1).

## 4 DISCUSSION

Generally, in describing the incidence of BCBM and identifying independent predictors of BM in the whole BC population, our results were in accordance with previous studies.^4^, ^5^, ^21^ In contrast to most of the published research on this topic, our work focused on identifying and analyzing the interaction effect among multiple predictors using a multiplicative interaction term and adjusting for all covariates.

BM was traditionally considered to occur in the terminal stage of BC progression and was associated with large tumor size and nodal involvement, referred to as the “linear progression model.”^23^ Another model of cancer spreading is called the “parallel progression model,”^32^, ^33^ which claims early tumor cell dissemination when the tumor is less advanced (e.g., when the tumor size is small) and independent development of the primary and metastatic tumors. In our analysis, the incidence of BM increased with a higher T stage whatever the molecular subtype, but the magnitude of the increase significantly differed in different molecular subtypes, and the sensitivity analysis excluding patients with unknown states of key factors confirmed this interactive effect between T stage and molecular subtypes. BC patients are predisposed to risk elevations for BM with a higher T stage in the general population except in the HR−/HER2− subgroup, where the T stage showed insignificant association with BM, showing that TNBC with small tumor size was as aggressive as that with large tumor size in the potential of developing BM. These data are not consistent with the linear progression model across all molecular subtypes but are consistent with the parallel progression model for TNBC. Our results indicated that in TNBC, the development of BM may arise from early dissemination of BC cells whatever the primary tumor size was, rather than being caused by late spillage of BC cells after the primary tumor comprises a lethal mass. Based on our analysis, early brain screening for TNBC patients should be encouraged.

In our study, although the incidence of BM increased with a higher *N* stage across all molecular subtypes in the whole BC population, the associations between the *N* stage and BM significantly differed by breast subtypes. In HR+/HER2− and HR−/HER2+ patients, the *N* stage had no significant relationship with BM, suggesting that lymph nodes may not be sources of BM in those patients, which may explain why removal of positive lymph nodes did not affect distant metastasis in some clinical trials.^34^, ^35^ By contrast, in HR−/HER2− patients, the higher the *N* stage, the higher the risk of developing BM, indicating that the positive lymph node status of TNBC patients may be a surrogate for inherent biologic aggressiveness in promoting BM. In light of our results, we consider for TNBC patients, the lymphatic metastatic route may be more dominant than the hematogenous route during the course of BM development. Unlike hematogenous dissemination which was known to be active in TNBC, the lymphatic metastatic route has been less studied in TNBC. The presence of lymphatic tissue within the central nervous system (CNS) remained undetected until 2015,^36^ raising the question of whether the CNS lymphatic pathway may be involved in the spreading of BC cells to the brain. Although the interactive effect between *N* stage and BM was questioned in the sensitivity analysis excluding patients with unknown states of key factors, we consider our results to be of concern and warrant further research.

For BM patients with only one ECM site, the association of ECM pattern with BM was significant only in the HR−HER2− subgroup for patients with only one ECM site. Among these patients, compared to those with bone metastasis, patients with lung metastasis had a higher risk of BM. These results indicate that brain‐specific metastasis may share more similarities with lung‐specific metastasis than with liver‐specific or bone‐specific metastases in TNBC patients. Evidence has shown the brain and the lung have functional and genetic similarities during metastasis. For example, Zhang et al.'s research found the microvasculature in the liver was fenestrated, whereas, in the brain and the lung, the vasculature was constituted by a continuous layer of endothelial cells with tight junctions, making the vascular barriers harder to overcome by metastatic cells in colonizing their target organs.^37^ Moreover, the JAK–STAT signaling pathway was enriched in both the brain and the lung metastasis.^37^ Bos et al. also designed a 17‐gene BM signature, in which about one‐third of the genes (e.g., COX2, EGFR ligands, etc.) were shared with an 18‐gene lung metastasis signature but not associated with relapse to the bones, liver, or lymph nodes.^38^ As such, once lung metastasis occurs for TNBC patients, the priority of brain screening should be raised, rather than waiting for the neurological symptoms to appear. In addition, because obtaining BM biopsies is difficult, lung biology may be an alternative, which is easier to perform while exhibiting possibly similar biological profiles to guide the selection of targeted therapies.

Overall, our study expanded upon the preliminary correlation of clinicopathological parameters with BCBM in finding molecular subtypes that may influence the associations of BM with certain risk factors. We found in TNBC patients, the development of BM was not driven by larger tumor size, which may be related to the lymphatic metastatic route, and those with lung metastasis are more susceptible to developing BM. In summary, TNBC had distinct metastatic propensities, which may be accounted for by the following possible explanations. Most TNBC cancers are characterized as basal‐like, in which a lack of association between tumor size and lymph node positivity was also reported.^39^, ^40^ Previous research indicated that basal‐like BC and nonbasal‐like BC arise from different breast progenitors^41^ and are completely distinct entities with separate metastatic pathways.^42^ As a consequence, the size‐nodes‐metastasis rule which applies in nonbasal‐like BC may be violated by basal‐like BC.^43^

Our study is novel in analyzing the interaction effect among multiple predictors and their relationships with BCBM using appropriate statistical analytical methods. Our data provide clinically useful information that could be used to identify BC patients who are more likely to develop BM and need early surveillance, which may impact the manner in which we approach brain screening and future targeted prevention strategies. A major strength of this study is that our findings about how BC molecular subtypes influence the impacts of tumor size, nodal status, and ECM patterns on incidence BM implied the specific biology of TNBC, offered clinical evidence for the parallel progression model and pathway overlap between brain and lung metastasis, and yielded new insight into the mechanisms of BCBM.

There were some limitations in this study. First, multi‐organ metastases can be diagnosed synchronously or metachronously, but the temporal orders of organ‐specific metastases were not included in the SEER database. To minimize bias caused by potential chronology in the metastatic processes, only patients with single‐organ ECM were selected to evaluate the relationship between BM and ECM patterns. Second, as a retrospective study, it was hard to avoid selection or information bias. Since detailed information on metastasis and molecular subtype was provided by the SEER database in 2010, we enrolled patients only between 2010 and 2018. Moreover, the majority of the included cases were Caucasian and black, so it is worth considering whether the results are applicable to other populations, especially in Asian cohorts.

## 5 CONCLUSION

T stage and ECM patterns had different associations with BM in different molecular subtypes. HR−/HER2− BC had distinct features on BM development, manifested as a lack of tumor size effect and is associated with lung metastasis. Therefore, more vigilance is required for HR−/HER2− BC patients regarding the detection of BM.

## AUTHOR CONTRIBUTIONS

**Bo Shen:** Conceptualization (equal); formal analysis (equal); investigation (equal); methodology (equal); software (equal); writing – original draft (equal). **Jieqing Li:** Conceptualization (equal); funding acquisition (equal); investigation (equal); validation (equal); writing – original draft (equal). **Mei Yang:** Formal analysis (equal); project administration (equal); writing – review and editing (lead). **Kangkang Liu:** Funding acquisition (equal); methodology (equal); writing – original draft (supporting). **Junsheng Zhang:** Formal analysis (equal); software (equal); writing – review and editing (supporting). **Weiping Li:** Data curation (equal); writing – review and editing (supporting). **Yi Zhang:** Writing – review and editing (supporting). **Kun Wang:** Conceptualization (equal); formal analysis (equal); funding acquisition (supporting); project administration (equal); supervision (equal); writing – review and editing (lead).

## FUNDING INFORMATION

KW received grants from the National Natural Science Foundation of China (82171898), Beijing Medical Award Foundation (YXJL‐2020‐0941‐0758), General Program of National Natural Science Foundation of Guangdong Province (2022A1515012277), and the “Climbing” Program from Guangdong Provincial People's Hospital (DFJH202109). MY and KL received grants from the National Natural Science Foundation of China (82072939 and 82003418). JL received grants from Xisike‐leader Oncology Research Foundation (Y‐2019AZQN‐0510). The funders did not have any role in the design of the study, the collection, analysis, and interpretation of the data; the writing of the manuscript; and the decision to submit the manuscript for publication. All researchers acted independently from the study sponsors in all aspects of the study.

## CONFLICT OF INTEREST

The authors declare that they have no conflict of interest.

## ETHICS APPROVAL STATEMENT

This research was based on publicly available data from the SEER database (https://seer.cancer.gov/), and a data use agreement was assigned. Analysis of de‐identified data from the SEER program was exempt from medical ethics review and no informed consent was required. All procedures performed in studies involving human participants were in accordance with the ethical standards of the 1964 Helsinki declaration and its later amendments or comparable ethical standards.

## Supporting information


Appendix S1

## Data Availability

The data underlying this article are available in the SEER database at https://seer.cancer.gov/.
