# Supplementary material for: Interactive effects of molecular subtypes with tumor size and extracranial metastatic pattern on risk of brain metastasis in breast cancer patients: A population‐based study
Source: Cancer Med. 2022 Nov 9;12(6):6547–57. doi: 10.1002/cam4.5425 (PMC10067112; doi:10.1002/cam4.5425)
Supplement: Supplementary file 1 — Appendix S1 [file CAM4-12-6547-s001.docx]

**Supplemental Table 1.** Univariate and multivariate logistic regression analysis predicting brain metastasis from breast cancer in all breast cancer patients*

| Variables | Univariate analysis | |  | Multivariate analysis | |
| --- | --- | --- | --- | --- | --- |
|  | OR (95% CI) | P value |  | OR (95% CI) | P value |
| Age |  | <0.001 |  |  | <0.001 |
| 20-39 | Reference |  |  | Reference |  |
| 40-59 | 0.871 (0.713-1.064) | 0.175 |  | 1.324 (1.074-1.634) | 0.009 |
| 60-79 | 0.763 (0.625-0.931) | 0.008 |  | 1.259 (1.020-1.555) | 0.032 |
| ≥80 | 0.516 (0.399-0.668) | <0.001 |  | 0.721 (0.550-0.945) | 0.018 |
| Sex |  | 0.004 |  |  | 0.334 |
| Female | Reference |  |  | Reference |  |
| Male | 1.779 (1.197-2.643) | 0.004 |  | 1.230 (0.809-1.870) | 0.334 |
| Race |  | <0.001 |  |  | 0.074 |
| White | Reference |  |  | Reference |  |
| Black | 1.672 (1.481-1.886) | <0.001 |  | 0.932 (0.819-1.061) | 0.286 |
| American Indian/ Alaska Native | 1.323 (0.781-2.243) | 0.298 |  | 0.939 (0.537-1.642) | 0.826 |
| Asian or Pacific Islander | 0.845 (0.706-1.011) | 0.066 |  | 0.785 (0.650-0.947) | 0.011 |
| Laterality |  | <0.001 |  |  | 0.792 |
| Left | Reference |  |  | Reference |  |
| Right | 1.022 (0.931-1.122) | 0.641 |  | 1.037 (0.941-1.144) | 0.462 |
| Bilateral | 19.522 (9.915-38.437) | <0.001 |  | 0.989 (0.483-2.023) | 0.976 |
| Unknown | 23.674 (18.489-30.315) | <0.001 |  | 1.129 (0.841-1.516) | 0.420 |
| Subtype |  | <0.001 |  |  | <0.001 |
| HR+/HER2- | Reference |  |  |  |  |
| HR+/HER2+ | 2.836 (2.503-3.213) | <0.001 |  | 1.510 (1.319-1.728) | <0.001 |
| HR-/HER2+ | 4.951 (4.299-5.701) | <0.001 |  | 2.201 (1.876-2.582) | <0.001 |
| HR-/HER2- | 3.124 (2.772-3.519) | <0.001 |  | 2.204 (1.920-2.529) | <0.001 |
| Histology |  | <0.001 |  |  | <0.001 |
| Ductal | Reference |  |  | Reference |  |
| Lobular | 0.573 (0.467-0.702) | <0.001 |  | 0.716 (0.577-0.889) | 0.002 |
| Mixed ductal and lobular | 0.583 (0.445-0.764) | <0.001 |  | 0.817 (0.618-1.080) | 0.156 |
| Mucinous | 0.415 (0.245-0.703) | 0.001 |  | 1.318 (0.762-2.280) | 0.323 |
| Tubular | <0.001 (NE) | 0.984 |  | <0.001 (NE) | 0.986 |
| Metaplastic | 1.630 (0.943-2.818) | 0.080 |  | 1.061 (0.592-1.900) | 0.842 |
| Micropapillary | 0.676 (0.281-1.628) | 0.382 |  | 1.042 (0.422-2.577) | 0.929 |
| Adenocarcinoma | 18.258 (15.205-21.923) | <0.001 |  | 1.475 (1.178-1.846) | 0.001 |
| Papillary | 0.525 (0.131-2.105) | 0.363 |  | 1.794 (0.436-7.378) | 0.418 |
| Medullary | 0.319 (0.045-2.266) | 0.253 |  | 0.704 (0.097-5.104) | 0.729 |
| Cribriform | 0.987 (0.317-3.070) | 0.982 |  | 3.308 (1.006-10.880) | 0.049 |
| Inflammatory | 8.413 (5.897-12.002) | <0.001 |  | 1.020 (0.693-1.500) | 0.920 |
| Others | 4.392 (3.698-5.216) | <0.001 |  | 1.274 (1.045-1.554) | 0.017 |
| Grade |  | <0.001 |  |  | <0.001 |
| 1 | Reference |  |  | Reference |  |
| 2 | 3.938 (3.074-5.045) | <0.001 |  | 1.650 (1.277-2.133) | <0.001 |
| ≥3 | 8.854 (6.947-11.285) | <0.001 |  | 1.868 (1.439-2.424) | <0.001 |
| Unknown | 25.429 (19.777-32.698) | <0.001 |  | 2.371 (1.801-3.122) | <0.001 |
| T stage |  | <0.001 |  |  | <0.001 |
| ≤1 | Reference |  |  | Reference |  |
| 2 | 3.473 (2.982-4.045) | <0.001 |  | 1.342 (1.138-1.584) | <0.001 |
| 3 | 9.031 (7.570-10.773) | <0.001 |  | 1.469 (1.204-1.794) | <0.001 |
| 4 | 34.041 (29.407-39.406) | <0.001 |  | 1.752 (1.466-2.094) | <0.001 |
| Unknown | 31.466 (26.640-37.165) | <0.001 |  | 2.133 (1.747-2.605) | <0.001 |
| N stage |  | <0.001 |  |  | <0.001 |
| 0 | Reference |  |  | Reference | <0.001 |
| 1-2 | 5.832 (5.202-6.539) | <0.001 |  | 1.411 (1.239-1.607) | <0.001 |
| 3 | 13.805 (11.819-16.125) | <0.001 |  | 1.615 (1.353-1.927) | <0.001 |
| Unknown | 26.725 (22.505-31.736) | <0.001 |  | 1.694 (1.385-2.073) | <0.001 |
| Patterns of extracranial metastasis |  | <0.001 |  |  | <0.001 |
| Bone(+) liver(-) lung(-) | Reference |  |  | Reference |  |
| Bone(-) liver(+) lung(-) | 0.824 (0.631-1.076) | 0.154 |  | 0.604 (0.459-0.793) | <0.001 |
| Bone(-) liver(-) lung(+) | 1.581 (1.323-1.889) | <0.001 |  | 1.168 (0.969-1.406) | 0.102 |
| Bone(+) liver(+) lung(-) | 1.923 (1.605-2.305) | <0.001 |  | 1.452 (1.205-1.749) | <0.001 |
| Bone(+) liver(-) lung(+) | 2.560 (2.193-2.988) | <0.001 |  | 2.162 (1.844-2.535) | <0.001 |
| Bone(-) liver(+) lung(+) | 2.622 (2.005-3.429) | <0.001 |  | 1.705 (1.293-2.250) | <0.001 |
| Bone(+) liver(+) lung(+) | 5.490 (4.689-6.428) | <0.001 |  | 4.075 (3.459-4.800) | <0.001 |
| Bone(-) liver(-) lung(-) | 0.015 (0.013-0.018) | <0.001 |  | 0.025 (0.021-0.029) | <0.001 |

*Adjusted for age, sex, race, laterality, subtypes, histology, grade, T stage, N stage, and patterns of extracranial metastasis.

**Supplemental Table 2.** Multivariate logistic regression analysis predicting brain metastasis from breast cancer according to breast subtypes in all breast cancer patients*

| Variables | OR (95% CI)* | | | | Interaction p value** |
| --- | --- | --- | --- | --- | --- |
|  | HR+/HER2- | HR+/HER2+ | HR-/HER2+ | HR-/HER2- |  |
| Age |  |  |  |  | 0.053 |
| 20-39 | Reference | Reference | Reference | Reference |  |
| 40-59 | 1.194 (0.848, 1.68) | 2.357 (1.407, 3.947) | 0.855 (0.542, 1.348) | 1.489 (0.948, 2.337) |  |
| 60-79 | 1.218 (0.867, 1.71) | 1.991 (1.178, 3.364) | 0.951 (0.598, 1.511) | 1.316 (0.835, 2.074) |  |
| ≥80 | 0.815 (0.546, 1.216) | 0.634 (0.283, 1.420) | 0.496 (0.234, 1.053) | 0.665 (0.370, 1.195) |  |
| Trend p | 0.012 | <0.001 | 0.250 | 0.002 |  |
| Sex |  |  |  |  | 0.570 |
| Female | Reference | Reference | Reference | Reference |  |
| Male | 1.002 (0.566, 1.771) | 1.270 (0.532, 3.031) | 1.047 (0.097, 11.285) | 3.191 (1.108, 9.187) |  |
| Trend p | 0.996 | 0.590 | 0.970 | 0.032 |  |
| Race |  |  |  |  | 0.240 |
| White | Reference | Reference | Reference | Reference |  |
| Black | 1.027 (0.846, 1.245) | 0.904 (0.663, 1.233) | 0.844 (0.582, 1.225) | 0.828 (0.640, 1.070) |  |
| American Indian/ Alaska Native | 0.516 (0.188, 1.417) | 1.668 (0.495, 5.615) | 1.003 (0.212, 4.749) | 1.900 (0.703, 5.136) |  |
| Asian or Pacific Islander | 0.696 (0.520, 0.932) | 1.085 (0.745, 1.581) | 0.737 (0.455, 1.193) | 0.667 (0.414, 1.074) |  |
| Trend p | 0.050 | 0.708 | 0.558 | 0.100 |  |
| Laterality |  |  |  |  | 0.169 |
| Left | Reference | Reference | Reference | Reference |  |
| Right | 1.005 (0.873, 1.158) | 1.111 (0.885, 1.394) | 1.013 (0.767, 1.338) | 1.075 (0.866, 1.335) |  |
| Bilateral | 0.291 (0.040. 2.148) | NA | 1.714 (0.422, 6.954) | 5.265 (1.591, 7.438) |  |
| Unknown | 1.087 (0.718, 1.645) | 0.909 (0.398, 2.076) | 1.655 (0.740, 3.704) | 1.212 (0.610, 2.409) |  |
| Trend p | 0.647 | 0.814 | 0.571 | 0.054 |  |
| Histology |  |  |  |  | 0.906 |
| Ductal | Reference | Reference | Reference | Reference |  |
| Lobular | 0.691 (0.539, 0.886) | 0.542 (0.260, 1.131) | 2.269 (0.960, 5.363) | 0.644 (0.272, 1.522) |  |
| Mixed ductal and lobular | 0.700 (0.481, 1.019) | 0.986 (0.538, 1.807) | 1.470 (0.637, 3.392) | 1.006 (0.428, 2.367) |  |
| Mucinous | 1.513 (0.851, 2.688) | NA | NA | 2.539 (0.252, 5.745) |  |
| Tubular | NA | NA | NA | NA |  |
| Metaplastic | 0.732 (0.098, 5.489) | NA | NA | 1.330 (0.714, 2.479) |  |
| Micropapillary | 1.389 (0.431, 4.480) | 1.707 (0.396, 7.352) | NA | NA |  |
| Adenocarcinoma | 1.269 (0.909, 1.773) | 1.154 (0.638, 2.088) | 1.839 (1.055, 3.205) | 1.895 (1.152, 3.118) |  |
| Papillary | 1.385 (0.191, 0.124) | 8.947 (0.911, 87.868) | NA | NA |  |
| Medullary | NA | NA | 8.687 (1.161, 64.987) | NA |  |
| Cribriform | 2.873 (0.678, 12.178) | 14.783 (1.402, 155.902) | NA | NA |  |
| Inflammatory | 0.599 (0.241, 1.488) | 0.856 (0.355, 2.064) | 1.483 (0.695, 3.165) | 1.349 (0.675, 2.697) |  |
| Others | 1.258 (0.932, 1.697) | 1.292 (0.786, 2.123) | 1.371 (0.784, 2.397) | 1.321 (0.897, 1.946) |  |
| Trend p | 0.023 | 0.341 | 0.350 | 0.644 |  |
| Grade |  |  |  |  | 0.180 |
| 1 | Reference | Reference | Reference | Reference |  |
| 2 | 1.606 (1.203, 2.145) | 1.128 (0.523, 2.431) | 1.469 (0.188, 11.497) | 1.188 (0.407, 3.467) |  |
| ≥3 | 1.973 (1.458, 2.670) | 1.328 (0.620, 2.843) | 1.435 (0.186, 11.100) | 1.122 (0.395, 3.187) |  |
| Unknown | 2.202 (1.592, 3.045) | 1.736 (0.779, 3.869) | 2.221 (0.283, 17.417) | 1.522 (0.520, 4.455) |  |
| Trend p | <0.001 | 0.128 | 0.146 | 0.345 |  |
| T stage |  |  |  |  |  |
| ≤1 | Reference | Reference | Reference | Reference | 0.002 |
| 2 | 1.55 (1.221, 1.968) | 0.979 (0.660, 1.452) | 1.410 (0.862, 2.305) | 0.993 (0.701, 1.407) |  |
| 3 | 1.641 (1.227, 2.194) | 1.185 (0.741, 1.894) | 1.187 (0.658, 2.141) | 1.300 (0.863, 1.958) |  |
| 4 | 1.816 (1.399, 2.358) | 1.605 (1.065, 2.418) | 1.733 (1.050, 2.858) | 1.402 (0.959, 2.052) |  |
| Unknown | 2.097 (1.575, 2.793) | 1.686 (1.048, 2.712) | 3.489 (2.030, 5.998) | 1.499 (0.940, 2.388) |  |
| Trend p | <0.001 | 0.009 | <0.001 | 0.126 |  |
| N stage |  |  |  |  |  |
| 0 | Reference | Reference | Reference | Reference | 0.002 |
| 1-2 | 1.172 (0.980, 1.401) | 1.383 (1.006, 1.902) | 1.258 (0.865, 1.830) | 2.575 (1.905, 3.482) |  |
| 3 | 1.227 (0.944, 1.596) | 1.308 (0.841, 2.032) | 1.757 (1.097, 2.816) | 3.674 (2.515, 5.367) |  |
| Unknown | 1.106 (0.827, 1.478) | 2.873 (1.810, 4.559) | 1.701 (0.956, 3.025) | 3.297 (2.043, 5.319) |  |
| Trend p | 0.311 | <0.001 | 0.072 | <0.001 |  |
| Patterns of extracranial metastasis |  |  |  |  | <0.001 |
| Bone(+) liver(-) lung(-) | Reference | Reference | Reference | Reference |  |
| Bone(-) liver(+) lung(-) | 0.949 (0.619, 1.455) | 0.554 (0.315, 0.975) | 0.525 (0.281, 0.981) | 0.506 (0.243, 1.056) |  |
| Bone(-) liver(-) lung(+) | 1.200 (0.897, 1.607) | 0.759 (0.448, 1.288) | 1.093 (0.633, 1.888) | 1.922 (1.302, 2.838) |  |
| Bone(+) liver(+) lung(-) | 1.844 (1.413, 2.407) | 0.979 (0.650, 1.473) | 1.333 (0.801, 2.219) | 1.597 (0.964, 2.648) |  |
| Bone(+) liver(-) lung(+) | 2.155 (1.751, 2.653) | 1.927 (1.333, 2.787) | 2.568 (1.442, 4.572) | 2.820 (1.820, 4.371) |  |
| Bone(-) liver(+) lung(+) | 2.252 (1.394, 3.638) | 1.795 (0.993, 3.248) | 2.050 (1.054, 3.987) | 1.531 (0.835, 2.805) |  |
| Bone(+) liver(+) lung(+) | 4.132 (3.267, 5.226) | 3.336 (2.350, 4.735) | 4.352 (2.649, 7.151) | 6.543 (4.238, 10.101) |  |
| Bone(-) liver(-) lung(-) | 0.016 (0.012, 0.020) | 0.022 (0.015, 0.032) | 0.039 (0.024, 0.065) | 0.064 (0.044, 0.093) |  |
| Trend p | <0.001 | <0.001 | <0.001 | <0.001 |  |

*Adjusted for age, sex, race, laterality, histology, grade, T stage, N stage, and patterns of extracranial metastasis.

**Adjusted for age, sex, race, laterality, histology, subtype, grade, T stage, N stage, and patterns of extracranial metastasis.

**Supplemental Table 3.** Multivariate logistic regression analysis predicting brain metastasis from breast cancer according to breast subtypes in breast cancer after excluding patients with unknown T stage, N stage, grade, or laterality, or with an imprecise classification of histological subtypes

| Variables | OR (95% CI)* | | | | Interaction p value** |
| --- | --- | --- | --- | --- | --- |
|  | HR+/HER2- | HR+/HER2+ | HR-/HER2+ | HR-/HER2- |  |
| Age |  |  |  |  | 0.136 |
| 20-39 | Reference | Reference | Reference | Reference |  |
| 40-59 | 1.402 (0.914, 2.150) | 1.771 (0.992, 3.161) | 0.693 (0.391, 1.227) | 1.389 (0.821, 2.350) |  |
| 60-79 | 1.358 (0.885, 2.082) | 1.558 (0.860, 2.824) | 0.828 (0.466, 1.470) | 1.093 (0.640, 1.866) |  |
| ≥80 | 0.853 (0.509, 1.430) | 0.267 (0.074, 0.959) | 0.437 (0.165, 1.162) | 0.838 (0.422, 1.664) |  |
| Trend p | 0.018 | 0.004 | 0.302 | 0.133 |  |
| Sex |  |  |  |  | 0.398 |
| Female | Reference | Reference | Reference | Reference |  |
| Male | 0.683 (0.299, 1.559) | 1.280 (0.440, 3.725) | NA | 4.147 (1.0761, 5.981) |  |
| Trend p | 0.365 | 0.651 | 0.998 | 0.039 |  |
| Race |  |  |  |  | 0.486 |
| White | Reference | Reference | Reference | Reference |  |
| Black | 1.045 (0.820, 1.333) | 0.956 (0.642, 1.424) | 0.861 (0.531, 1.395) | 0.780 (0.568, 1.072) |  |
| American Indian/ Alaska Native | 0.778 (0.241, 2.507) | 0.752 (0.098, 5.786) | 1.656 (0.353, 7.767) | 2.559 (0.853, 7.678) |  |
| Asian or Pacific Islander | 0.820 (0.579, 1.161) | 1.044 (0.641, 1.702) | 0.640 (0.323, 1.265) | 0.507 (0.256, 1.001) |  |
| Trend p | 0.640 | 0.983 | 0.501 | 0.030 |  |
| Laterality |  |  |  |  | 0.776 |
| Left | Reference | Reference | Reference | Reference |  |
| Right | 1.097 (0.920, 1.309) | 1.270 (0.950, 1.696) | 0.840 (0.590, 1.194) | 1.062 (0.816, 1.381) |  |
| Bilateral | NA | NA | 1.267 (0.131, 12.273) | 3.323 (0.610, 8.418) |  |
| Trend p | 0.586 | 0.271 | 0.599 | 0.367 |  |
| Histology |  |  |  |  | 0.970 |
| Ductal | Reference | Reference | Reference | Reference |  |
| Lobular | 0.763 (0.557, 1.045) | 0.905 (0.386, 2.123) | 2.602 (0.931, 7.270) | 0.548 (0.167, 1.801) |  |
| Mixed ductal and lobular | 0.649 (0.411, 1.027) | 1.160 (0.590, 2.283) | 1.426 (0.535, 3.802) | 0.838 (0.294, 2.384) |  |
| Mucinous | 1.972 (1.017, 3.825) | NA | NA | NA |  |
| Tubular | NA | NA | NA | NA |  |
| Metaplastic | 1.055 (0.135, 8.253) | NA | NA | 1.543 (0.746, 3.191) |  |
| Micropapillary | 0.522 (0.071, 3.826) | 1.065 (0.137, 8.292) | NA | NA |  |
| Adenocarcinoma | 2.982 (1.537, 5.786) | 5.832 (1.672, 0.365) | 0.743 (0.089, 6.176) | 4.236 (1.936, 9.268) |  |
| Papillary | NA | 17.614 (1.352, 29.797) | NA | NA |  |
| Medullary | NA | NA | 14.933 (1.971, 113.133) | NA |  |
| Cribriform | 4.154 (0.951, 8.165) | 29.102 (2.842, 98.195) | NA | NA |  |
| Inflammatory | 0.663 (0.239, 1.842) | 1.042 (0.356, 3.052) | 2.367 (0.951, 5.893) | 1.451 (0.635, 3.318) |  |
| Trend p | 0.006 | 0.044 | 0.256 | 0.141 |  |
| Grade |  |  |  |  | 0.420 |
| 1 | Reference | Reference | Reference | Reference |  |
| 2 | 1.783 (1.271, 2.501) | 2.302 (0.757, 6.997) | 1.260 (0.159, 9.996) | 1.594 (0.369, 6.889) |  |
| ≥3 | 2.110 (1.478, 3.012) | 2.386 (0.788, 7.226) | 1.026 (0.131, 8.050) | 1.359 (0.321, 5.752) |  |
| Trend p | <0.001 | 0.306 | 0.587 | 0.588 |  |
| T stage |  |  |  |  | 0.035 |
| ≤1 | Reference | Reference | Reference | Reference |  |
| 2 | 1.522 (1.135, 2.040) | 0.839 (0.514, 1.370) | 1.476 (0.828, 2.632) | 0.966 (0.633, 1.476) |  |
| 3 | 1.627 (1.144, 2.312) | 1.155 (0.656, 2.034) | 1.044 (0.525, 2.076) | 1.134 (0.686, 1.874) |  |
| 4 | 1.946 (1.412, 2.683) | 1.488 (0.894, 2.476) | 1.349 (0.729, 2.496) | 1.377 (0.862, 2.199) |  |
| Trend p | 0.001 | 0.031 | 0.431 | 0.269 |  |
| N stage |  |  |  |  | 0.157 |
| 0 | Reference | Reference | Reference | Reference |  |
| 1-2 | 1.042 (0.836, 1.300) | 1.421 (0.944, 2.138) | 1.031 (0.650, 1.636) | 2.438 (1.685, 3.530) |  |
| 3 | 1.050 (0.767, 1.438) | 1.247 (0.719, 2.164) | 1.427 (0.801, 2.544) | 3.461 (2.190, 5.470) |  |
| Trend p | 0.927 | 0.228 | 0.354 | <0.001 |  |
| Patterns of extracranial metastasis |  |  |  |  | <0.001 |
| Bone(+) liver(-) lung(-) | Reference | Reference | Reference | Reference |  |
| Bone(-) liver(+) lung(-) | 0.772 (0.443, 1.344) | 0.513 (0.239, 1.100) | 0.344 (0.135, 0.878) | 0.395 (0.151, 1.038) |  |
| Bone(-) liver(-) lung(+) | 1.141 (0.801, 1.626) | 0.593 (0.286, 1.230) | 1.419 (0.731, 2.755) | 1.742 (1.078, 2.816) |  |
| Bone(+) liver(+) lung(-) | 1.645 (1.156, 2.340) | 1.004 (0.584, 1.726) | 2.065 (1.085, 3.931) | 2.044 (1.145, 3.649) |  |
| Bone(+) liver(-) lung(+) | 1.970 (1.511, 2.567) | 2.393 (1.501, 3.816) | 3.309 (1.636, 6.691) | 3.345 (1.966, 5.692) |  |
| Bone(-) liver(+) lung(+) | 2.386 (1.341, 4.248) | 2.365 (1.170, 4.782) | 1.722 (0.687, 4.318) | 1.160 (0.514, 2.619) |  |
| Bone(+) liver(+) lung(+) | 4.050 (2.992, 5.482) | 4.056 (2.582, 6.373) | 4.039 (2.114, 7.716) | 6.227 (3.658, 10.602) |  |
| Bone(-) liver(-) lung(-) | 0.010 (0.007, 0.013) | 0.016 (0.010, 0.027) | 0.023 (0.012, 0.045) | 0.043 (0.027, 0.068) |  |
| Trend p | <0.001 | <0.001 | <0.001 | <0.001 |  |

*Adjusted for age, sex, race, laterality, histology, grade, T stage, N stage, and patterns of extracranial metastasis.

**Adjusted for age, sex, race, laterality, histology, subtype, grade, T stage, N stage, and patterns of extracranial metastasis.

**Supplemental Table 4.** Multivariate logistic regression analysis predicting brain metastasis from breast cancer according to breast subtypes in breast cancer patients with a single extracranial metastatic site*

| Variables | OR (95% CI)* | | | | Interaction p value** |
| --- | --- | --- | --- | --- | --- |
|  | HR+/HER2- | HR+/HER2+ | HR-/HER2+ | HR-/HER2- |  |
| Age |  |  |  |  | 0.606 |
| 20-39 | Reference | Reference | Reference | Reference |  |
| 40-59 | 1.249 (0.743, 2.100) | 2.444 (1.029, 5.807) | 4.050 (0.943, 17.397) | 2.221 (0.926, 5.328) |  |
| 60-79 | 1.420 (0.851, 2.371) | 2.836 (1.194, 6.740) | 4.985 (1.157, 21.471) | 1.692 (0.701, 4.083) |  |
| ≥80 | 0.601 (0.318, 1.134) | 1.145 (0.355, 3.699) | 2.713 (0.474, 15.535) | 0.892 (0.318, 2.501) |  |
| Trend p | 0.002 | 0.029 | 0.133 | 0.023 |  |
| Sex |  |  |  |  | 0.993 |
| Female | Reference | Reference | Reference | Reference |  |
| Male | 0.710 (0.260, 1.941) | 0.511 (0.067, 3.868) | NA | NA |  |
| Trend p | 0.505 | 0.516 | 0.999 | 0.999 |  |
| Race |  |  |  |  | 0.895 |
| White | Reference | Reference | Reference | Reference |  |
| Black | 0.932 (0.683, 1.272) | 1.305 (0.806, 2.111) | 1.470 (0.822, 2.627) | 0.828 (0.539, 1.270) |  |
| American Indian/ Alaska Native | 0.855 (0.207, 3.539) | NA | NA | 1.762 (0.378, 8.227) |  |
| Asian or Pacific Islander | 0.701 (0.440, 1.117) | 1.125 (0.565, 2.240) | 0.937 (0.382, 2.302) | 0.588 (0.230, 1.503) |  |
| Trend p | 0.505 | 0.753 | 0.611 | 0.487 |  |
| Laterality |  |  |  |  | 0.713 |
| Left | Reference | Reference | Reference | Reference |  |
| Right | 1.123 (0.904, 1.396) | 1.010 (0.690, 1.479) | 0.804 (0.494, 1.309) | 1.102 (0.763, 1.590) |  |
| Bilateral | NA | 00 | 5.572 (0.570, 54.434) | 00 |  |
| Unknown | 0.633 (0.310, 1.291) | 0.705 (0.212, 2.344) | 0.888 (0.203, 3.886) | 0.485 (0.129, 1.825) |  |
| Trend p | 0.363 | 0.951 | 0.356 | 0.656 |  |
| Histology |  |  |  |  | 0.963 |
| Ductal | Reference | Reference | Reference | Reference |  |
| Lobular | 0.622 (0.437, 0.887) | 0.211 (0.050, 0.889) | 1.274 (0.346, 4.694) | 0.245 (0.033, 1.828) |  |
| Mixed ductal and lobular | 0.529 (0.287, 0.978) | 1.440 (0.639, 3.245) | 0.645 (0.084, 4.946) | 1.675 (0.569, 4.931) |  |
| Mucinous | 2.133 (0.904, 5.033) | NA | NA | NA |  |
| Tubular | NA | NA | NA | NA |  |
| Metaplastic | NA | NA | NA | 0.772 (0.229, 2.601) |  |
| Micropapillary | 0.922 (0.124, 6.857) | 1.983 (0.236, 16.701) | NA | NA |  |
| Adenocarcinoma | 1.175 (0.673, 2.048) | 1.276 (0.528, 3.088) | 1.422 (0.486, 4.158) | 3.077 (1.447, 6.543) |  |
| Papillary | 3.186 (0.420, 5.408) | NA | NA | NA |  |
| Medullary | NA | NA | NA | NA |  |
| Cribriform | NA | NA | NA | NA |  |
| Inflammatory | 0.975 (0.348, 2.733) | 0.455 (0.059, 3.474) | 0.983 (0.201, 4.801) | 1.416 (0.402, 4.995) |  |
| Others | 1.405 (0.907, 2.178) | 0.841 (0.335, 2.110) | 1.560 (0.608, 4.004) | 2.422 (1.337, 4.388) |  |
| Trend p | 0.079 | 0.637 | 0.994 | 0.145 |  |
| Grade |  |  |  |  | 0.110 |
| 1 | Reference | Reference | Reference | Reference |  |
| 2 | 1.590 (1.001, 2.527) | 0.416 (0.152, 1.143) | NA | NA |  |
| ≥3 | 2.128 (1.318, 3.437) | 0.510 (0.188, 1.381) | NA | NA |  |
| Unknown | 2.168 (1.312, 3.582) | 0.826 (0.289, 2.364) | NA | NA |  |
| Trend p | 0.004 | 0.064 | 0.633 | 0.923 |  |
| T stage |  |  |  |  | 0.092 |
| ≤1 | Reference | Reference | Reference | Reference |  |
| 2 | 1.129 (0.784, 1.623) | 0.673 (0.368, 1.232) | 0.415 (0.177, 0.973) | 0.747 (0.405, 1.376) |  |
| 3 | 1.295 (0.860, 1.948) | 0.668 (0.324, 1.377) | 0.654 (0.264, 1.617) | 0.701 (0.358, 1.374) |  |
| 4 | 1.306 (0.895, 1.907) | 0.946 (0.513, 1.745) | 0.692 (0.325, 1.474) | 0.564 (0.303, 1.048) |  |
| Unknown | 1.310 (0.847, 2.026) | 0.858 (0.403, 1.829) | 1.258 (0.544, 2.910) | 0.686 (0.312, 1.508) |  |
| Trend p | 0.574 | 0.552 | 0.100 | 0.483 |  |
| N stage |  |  |  |  | 0.056 |
| 0 | Reference | Reference | Reference | Reference |  |
| 1-2 | 0.992 (0.761, 1.292) | 1.069 (0.645, 1.771) | 0.753 (0.406, 1.394) | 1.363 (0.847, 2.194) |  |
| 3 | 1.179 (0.818, 1.701) | 0.921 (0.452, 1.874) | 0.771 (0.357, 1.666) | 1.286 (0.691, 2.390) |  |
| Unknown | 0.903 (0.565, 1.442) | 2.416 (1.154, 5.059) | 1.323 (0.512, 3.419) | 1.353 (0.600, 3.051) |  |
| Trend p | 0.703 | 0.082 | 0.565 | 0.640 |  |
| Patterns of extracranial metastasis |  |  |  |  | 0.013 |
| Bone(+) liver(-) lung(-) | Reference | Reference | Reference | Reference |  |
| Bone(-) liver(+) lung(-) | 0.929 (0.605, 1.427) | 0.557 (0.315, 0.986) | 0.461 (0.244, 0.870) | 0.455 (0.219, 0.948) |  |
| Bone(-) liver(-) lung(+) | 1.186 (0.881, 1.597) | 0.751 (0.440, 1.284) | 0.800 (0.456, 1.404) | 1.936 (1.300, 2.882) |  |
| Trend p | 0.473 | 0.103 | 0.057 | <0.001 |  |

*Adjusted for age, sex, race, laterality, histology, grade, T stage, N stage, and patterns of extracranial metastasis.

**Adjusted for age, sex, race, laterality, histology, subtype, grade, T stage, N stage, and patterns of extracranial metastasis.
